# The “Sandwich” System: A Potential Solution for Protecting Overwintering *Cornu aspersum* Snails Reared in Semi-Intensive Heliciculture Farms in Colder Climates

**DOI:** 10.3390/ani11051420

**Published:** 2021-05-15

**Authors:** Dan Manea, Anișoara Aurelia Ienciu, Ramona Ștef, Ioan Peț, Laura Șmuleac, Ioana Grozea, Alin Cărăbeț, George Andrei Drăghici, Dragoș Vasiles Nica

**Affiliations:** 1 Faculty of Agriculture, Banat’s University of Agricultural Sciences and Veterinary Medicine “King Mihai I of Romania”, Calea Aradului 119, 300645 Timişoara, Romania; manea_dn@yahoo.com (D.M.); ienciuani@yahoo.com (A.A.I.); laurasmuleac@yahoo.com (L.Ș.); ioana_entomol@yahoo.com (I.G.); alin70599@yahoo.co.uk (A.C.); 2 Faculty of Bioengineering of Animal Resources, Banat’s University of Agricultural Sciences and Veterinary Medicine “King Mihai I of Romania”, Calea Aradului 119, 300645 Timişoara, Romania; ioanpet@eurofins.com; 3 Faculty of Pharmacy, “Victor Babes” University of Medicine and Pharmacy Timisoara, Piaţa Eftimie Murgu 2, 300041 Timisoara, Romania; draghici.george-andrei@umft.ro

**Keywords:** snail, farming, winterprotection, hibernation, overwintering, *Cornu aspersum*

## Abstract

**Simple Summary:**

The Italian semi-intensive (ISISF) technology is widely used for rearing the Mediterranean snail, *Cornu aspersum*. It relies on protecting overwintering specimens with Lutrasil frost cloth (LFC). This approach yielded elevated mortalities in Romanian snail farms. We aimed to develop a simple and effective system for protecting overwintering *C. aspersum* adults in colder climates. This three-year, three-phase experiment investigated selected behavioral aspects and thermal protection efficiency of different protective structures. Mature gastropods in preparation for hibernation exhibited a significant preference for wood and ridge-tile micro shelters. Soil texture significantly influenced the burrowing behavior, but not the burrowing depth. The structure soil/LFC/straw/10-cm air cushion/high-density polyethylene (HDPE)—the “sandwich” system—was selected to be used as a protective system. Under farm conditions, adult snails tended to hibernate clustered together, attached to the lower surface of micro shelters. The “sandwich” system coupled with using ridge-tile/wood micro shelters resulted in significantly higher survival thanthe sole use of LFC. Predator occurrence appeared to exert a minor effect on snail survival. These data render the “sandwich” system a potential solution for overwintering *C. aspersum* breeders in colder climates.

**Abstract:**

(1) Background: Hibernation in pens covered with LFC was associated with high mortality of *C. aspersum* snails in Romanian snail farms. This three-year study aimed to develop a simple, but effective system for protecting breeders in colder climates. (2) Methods: The first phase investigated the (pre)hibernal burrowing behavior and the overwintering habitat choice. Protective structures based on straw, LFC, and/or HDPE were tested at pilot level (no snails). The most suitable system was applied under farm conditions. (3) Results: Wood and ridge-tile micro shelters were significantly preferred to corrugated iron micro shelters. Burrowing specimens acted as shallow-burrowers, and this behaviorwas significantly more common for looser soils. All pilot systems displayed significantly higher thermal protection efficiency compared to the sole use of LFC. The balance between straw moistening and thermal protection favored using structure soil/LFC/straw/10-cm air cushion/HDPE. Its use yielded significantly higher survival compared to the sole use of LFC. Most hibernating snails clustered together in large groups, attached on the lower surface of micro shelters. Predator occurrence appeared to marginally affect overwintering survival. (4) Conclusions: The “sandwich” system could be an effective solution for overwintering mature *C. aspersum* snails in colder climates.

## 1. Introduction

In the last decades, small animal farming has attracted increasing research and economic interest as a potential alternative to the husbandry of large animal farms (e.g., pigs, cattle, poultry). Since rearing small animals requires less water, space, and financial investment while also generating lower amounts of methane, nitrous oxide, and carbon dioxide than large livestock farming, these farms are more environmentally friendly and easier to implement and manage. Taken together with the reorientation of human nutrition towards a healthy diet, these advantages have encouraged the development of more sustainable, safer, and less costly farming alternatives, including the production of animal proteins from nontraditional farmed animals, such as terrestrial snails or insects [1].

Used as food since prehistoric times, the snail meat is highly prized in the modern diet due to its high content of proteins rich in essential aminoacids (12.9%), low content of fat (0.6–1.5%, mainly polyunsaturated), and elevated content of minerals (about 2.4 g of Ca, Fe, P, and Cu per 100 g) [2,3,4,5]. Rearing of edible land snails (heliciculture) in open pastures is routinely used in Mediterranean countries (e.g., Italy, Spain, France), with the brown garden snail, *Cornu aspersum* (Müller, 1774; syn. *Helix aspersa*, *Cantareus aspersus*, or *Cryptomphalus aspersus*), being the main species of commercial interest. Native to the Mediterranean Basin and parts of Western Europe [6], this fast-growing gastropod is more prolific and adaptable to different environmental conditions than other land snails of economic importance [7,8]. This renders farming of *C. aspersum* easier, profitable, and less risky [9,10].

The rearing of *C. aspersum* using the Italian semi-intensive snail farming (ISISF) system is based on the characteristics of the life cycle of this species, with the snails being bred, grown, and fattened on open pastures of fresh vegetables in free-range pens in a natural environment [11]. The breeding herd, which is made up of sexually mature individuals aged 8 to 12 months, is introduced into breeding pens during late spring to early summer. After three months, these snails are transferred into newly established breeding pens, where they overwinter together with their juveniles. The cycle is resumed during the next spring, with the breeders and their adult offspring being sold in the autumn of the same year [8].

Driven by a significant share of economically underexploited arableareas and low labor costs compared to the Western European countries, this method of snail farming has enjoyed significant growth in the former communist countries of Central and Eastern Europe during the first decade of this millennium [7,8]. For example, over 200 heliciculture farms were using the ISISF system in 2006 only in Romania [4]. Following this technology, the Lutrasil frost cloth (LFC) between 18 and 30 g per square meter (g/m^2^) is used to protect *C. aspersum* during hibernation [8]. It is applied after cutting the vegetation off to a height of 10 to 20 cm. However, this method could be ineffective during the colder winters specific to the humid continental climate of Central Eastern Europe, potentially leading to increased mortalities among overwintering snails. In fact, death rates of mature *C. aspersum* snails during the winter of 2005/2006 have exceeded 70% in most snail farms from Romania using the standard ISISF system [4,12]. This was a critical issue given the central role of breeding herd survival in successful snail farming [8,13].

Like other ectotherm animals, terrestrial gastropods spend the winter in hibernation—a physiological status of obligate dormancy during which metabolism decreases to subsistence level [14]. Although very successful in colonizing new areas [6], *C. aspersum* is a partially freezing tolerant species [15], for which in Central and Eastern Europe only a few stable populations in restricted areas have been reported [16,17,18]. The gregarious behavior of this land snail is particularly apparent during the winter months when specimens of different sizes tend to aggregate in sheltered sites, such as under stones and decaying wood, or in the crevices of stone walls [9]. However, there is evidence that *C. aspersum* can burrow itself into soil or leaf litter to avoidcold winter weather [9,19], but little knowledge exists about the burrowing depth. The texture is an important edaphic factor affecting snail distribution in terrestrial environments [20,21], yet limited information is available about its effect on burrowing behavior. Moreover, no study has investigated these habits under farm conditions.

Here, we aimed at developing a simple, cost-effective, but efficient system for protecting mature brown garden snails, *C. aspersum*, during the winter months. We first tested different types of protective structures built from materials with good thermal insulation properties that were easily available and cost-effective, more precisely high-density polyethylene (HDPE), straw, and LFC [22,23,24]. The most suitable system was then applied under real farm conditions. The aforementioned habits of (pre)overwintering snails were also studied given their relevance for mimicking the proper environment for snail hibernation and choosing the proper moment when to install this winter protection system. Results were obtained from experiments conducted in seven snail farms in three consecutive years and cumulated with additional field observations performed in the other six farms.

## 2. Materials and Methods

### 2.1. C. aspersum (Pre)hibernal Behavior

On 4 October 2004, three areas of 2.5 m^2^ each were fenced inside a breeding pen from a snail farm located in the village of Peciu Nou (Timiş county; latitude 45°36′ N, longitude 21°31′ E). After collecting the snails inside them, corrugated roofing sheets (rectangles of up to 0.25 m^2^), ridge tiles, or wooden pallets (325 × 265 × 70 mm) were put into each enclosure before populating it with 50 adult specimens of *C. aspersum*. The snails were collected after three weeks to determine their preference for different types of micro shelters.

The burrowing behavior was investigated in two snails farms with different soil textures, that is a commercial heliciculture farm from the village of Urecheşti (Gorj county; latitude 44°57′ N, longitude 23°16′ E) having a medium-sandy clay soil; and another farm, located in the village of Banloc (Timiş county; latitude 45°38′ N, longitude 21°13′ E) and having medium clay soil. Three areas of 5 m^2^ each were randomly fenced inside the breeding pens of each farm, and the gastropods inside were transferred to other pens. Micro shelters (used wooden pallets, old wood slabs) were then placed on the ground to cover at least half of each fenced area. After being populated with 100 adult individuals on 19 October 2005, the enclosures were covered with LFC (23 g/m^2^). The snails were collected on 1 December 2005.

### 2.2. Identification of Protection System (Pilot Level)

This study was conducted in March 2006 in the village of Sâmbata de Sus (Braşov county; latitude 45°45′ N, longitude 24°91′ E). Being located at the foot of the highest mountain peak from Romania (Moldoveanu, 2544 m), this site has cold to very cold winters, with frequent temperatures below −15 °C [24]. The thermal efficiencies of the following protective systems were tested: (i) S1, soil/LFC; (ii) S2, soil/straw/LFC; (iii) S3, soil/LFC/straw/HDPE; (iv) S4, soil/LFC/straw/10-cm air cushion/HDPE. In all cases, we used an LFC of 23 g/m^2^, and the protected surface was 10 m^2^ (5 × 2 m). During this one-week experiment, the inside and outside temperatures were measured daily with an electronic thermometer at 3 am. Measurements were performed at 5 cm above the soil surface for three points, i.e., both extremities of the structure and the middle of the structure. The average temperature during a 15 min. interval was recorded for each point, and only the mean value was used for statistical analysis.

Apart from temperature, moisture levels are important for the viability of *C. aspersum*. For example, this gastropod species is active at temperatures between 7 °C and 21 °C and an elevated humidity of 75–90% [8,9]. Under unfavorable conditions (e.g., low temperature and/or low humidity), its activity decreases with snails being able to remain dormant for several months [6,8,11]. Importantly, *C. aspersum* exhibits a moderately enhanced cold hardiness concerning hibernation, and it cannot survive long periods of frost [6,15], such as those often encountered during winter under the humid temperate continental climate conditions from Romania [25]. Since prolonged frosts can induce the freezing of moist straw [23], we considered straw moisture an important parameter for successfully developing effective systems for protecting overwintering *C. aspersum* snails. This parameter was estimated empirically by taking a bundle of straw out and twisting/squeezing it in the hand. The following classes of straw moisture were considered: (i) completely dry straw; (ii) slight straw moistening; (iii) moderate straw moistening; (iv) high straw moistening (wet straw).

### 2.3. Testing of Protective Structure under Realistic Farm Conditions

The concept developed based on data derived from the pilot phase (i.e., the “sandwich” system) was tested under realistic field conditions in three snails farms located in Cugir (Alba county; latitude 45°83′ N, longitude 23°36′ E), Ezeriş (Caraş-Severin county; latitude 45°23′ N; longitude, 21°52′ E), and Muntenii de Sus (Vaslui county; latitude 46°70′ N, longitude: 27°76′ E). These farms were populated with mature snails aged 12–16 months between May and June 2006. The initial herd was purchased from the International Snail Farming Institute (Cherasco, Italy). Following the standard workflow of the ISISF system, surviving specimens were transferred into newly established breeding pens about three months after populating the farm (from July to September 2006), with juveniles remaining in the first established breeding pens.

The “sandwich” system was used only for protecting the latter pens. It was installed in October/November 2006 when night temperatures were constantly below 5 °C for three consecutive days, and mature snails were observed starting to prepare for hibernation. Before this activity, the vegetation in the pens was cutoff to a height of 10 cm. The protective system was dismantled in March/April 2007 on dry weather when night temperatures constantly remained above 5 °C for at least four days. The thickness of the wheat straw layer was 10 cm for the farms located in Cugir and Ezeriş and 15 cm for the farm located in Muntenii de Sus. The rationale behind this was that the latter farm was in Moldova, a region influenced by Scandinavian-Baltic weather and with colder winters compared to the two former sites [25]. At this site, a supplementary breeding pen was also established in September 2006 and populated with 1000 sexually mature *C. aspersum* snails. This reference enclosure was covered with LFC (23 g/m^2^) during the winter months.

After dismantling the protective system, the breeding pens were watered daily for three consecutive days to promote spring arousal. All snails (alive individuals and empty shells separately) were collected, and the living specimens were transferred into new breeding pens. The overwintering success was assessed based on the survival rate. Survival was expressed as a percentage and was calculated as the ratio of the number of living snails post-hibernation to the number of living specimens post-hibernation. We assigned a probable death cause to all collected shells. We did not know the actual date and cause of death but estimated them using the following criteria: (i) presence of the body inside the shell; (ii) extent of the weathering of shell nacreous interior after death; (iii) presence of shell smashed or with holes in the spire.

We also determined the thermal efficiency of the “sandwich” system. Measurements were performed in triplicate within a breeding pen from the commercial heliciculture farm in Muntenii de Sus. The measurement protocol was similar tothat used during pilot testing. Measurement time points were chosen to reflect the thermal behavior of the “sandwich” system under both cold weather (during the frozen period, i.e., December–February) and mild weather conditions (before spring, i.e., end of February–March).

Throughout this time, the (pre)hibernal behavior of mature snails was monitored in six other heliciculture farms. Although investigating survival of juveniles and their (pre)hibernal behavior were not within the scope of the present work, general observations were, however, made. Taken together, these latter data should provide us with a more detailed understanding of behavioral aspects of (pre)overwintering *C. aspersum* in outdoor snail farms.

### 2.4. Statistical Analysis

The snails were stratified into specimens attached to the lower (inner) surface of the micro shelters and unattached specimens. We conducted a Chi^2^ test on a 3 × 2 contingency table to determine the differences in the distribution of these classes across different types of micro shelters. In the case of significant results, posthoc analysis with Chi^2^ tests based on 2 × 2 contingency tables were applied to find which pairs of cells are significantly different. Paired comparisons with Chi^2^ tests based on 2 × 2 contingency tables were next run to assess the effect of soil texture on burrowing behavior characterized across three distinct parameters. To this end, the snails were first categorized into burrowing specimens and non-burrowing specimens. The burrowing gastropods were next classified into individuals burrowing within the top 5 cm soil layer and individuals burrowing deeper into the soil. After this, the non-burrowing snails were split into specimens attached to the lower surface of micro shelters and specimens lying on the ground (unattached).

A Friedman test was run on temperature data measured at a pilot level to identify the ability of different protection structures to preserve the soil thermal inertia during wintertime. Post hoc Wilcoxon tests against the outdoor temperature were conducted in the case of significant differences. The thermal efficiency of the protection system selected to be used was next analyzed under realistic field conditions. A Wilcoxon test was performed to compare the inside and outside air temperatures. Next, we determined differences in survival rates of *C. aspersum* adults protected overwinter with the “sandwich” system (Cugir, Ezeriş, Muntenii de Sus) and those protected using only LFC (Muntenii de Sus) by conducting a Chi^2^ test on a 4 × 2 contingency table. In the case of significant differences, posthoc analyses were performed with Chi^2^ tests based on 2 × 2 contingency tables against the later variable. Finally, we applied a Chi^2^ test based on 4 × 4 contingency table on the distribution of shell condition classes to investigate differences in potential death causes across different locations. For significant differences, the frequencies observed for the reference pen were used as a benchmark for post hoc comparisons with Chi^2^ tests based on 2 × 2 contingency tables. All statistical analyses were performed using Statistica version 8 (StatSoft Inc., Tulsa, OK, USA). In all of the cases, a *p* value less than 0.05 was considered significant. To reduce the error in approximation, we adjusted the values of Chi^2^ according to Yates’s correction for continuity and applied post hoc Wilcoxon tests with Bonferroni correction.

## 3. Results

### 3.1. C. aspersum (Pre)hibernal Behavior

Data related to the preference of mature *C. aspersum* snails for different types of micro shelters are shown in Table 1. Statistical analysis yielded significant differences in the distribution of attached and unattached specimens across micro shelters tested (chi^2^ test, *p* < 0.001). Post hoc testing revealed a significantly higher tendency of snails to avoid corrugated iron as a shelter when compared to ridge tiles and wooden pallets (Table 1). However, no significant differences were identified between the two former types of micro shelters (Table 1).

The distribution of adult snails across different classes of burrowing behavior isgiven in Table 2. At least two-thirds of individuals did not burrow into the soil, irrespective of soil texture (Table 2). For those that did burrow, a significantly (two-fold) higher proportion burrowed in the medium-sandy clay soil (ST1) than in the medium clay soil (ST2) (Table 2). However, no significant differences were found between different classes related to burrowing depth, with the vast majority of snails only burrowing down to 5 cm regardless of soil texture (Table 2). Similar results were obtained when analyzing the distributions of non-burrowed specimens (Table 2). The frequency of gastropods attached to the lower part of micro shelters was comparable to that observed in the previous experiment (Table 1 and Table 2).

### 3.2. Identification of Protection System (Pilot Level)

**Figure 1:**
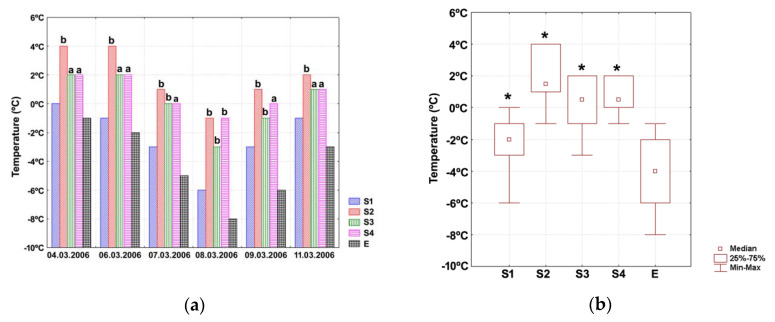
(**a**) Absolute temperature values measured at selected time points for different protective structures. Marked columns (^a,b^) indicate the degree of straw moistening (^a^—slight straw moistening, ^b^—moderate straw moistening). S1, soil/LFC; S2, soil/straw/LFC; S3, soil/LFC/straw/HDPE; S4, soil/LFC/straw/10-cm air cushion/HDPE; E, no protection structure (soil). (**b**) Median temperatures for different protective structures. Data are shown as medians (point), with lower and upper quartiles (box) and minimum and maximum values (error bars). Marked boxes (*) indicate significant differences than the reference group (E) (Wilcoxon tests, ***—*p* ≤ 0.001, **—*p* ≤ 0.01, *—*p* ≤ 0.05).

During the one-week testing period, the night temperature measured at 5 cm above the soil surface ranged between 0 °C and −8 °C, whereas the day temperature varied between −1 °C and +6 °C. No days with precipitation (snow, rain) were recorded throughout the experimental period, except for the second time point when moderate snowfall occurred. Temperatures measured at different time points inside and outside protection systems are given in Figure 1a. The corresponding median values, with lower and upper quartiles and minimum and maximum values, are illustrated in Figure 1b.

Significant differences existed in temperature medians (Friedman’s test *p* = 0.001). Post hoc analyses showed that temperatures inside all protective systems were significantly higher than the ambient temperature (Figure 1b), with the highest value being recorded for the structure S2 (soil/straw/LFC). Snow melting resulted into moderate straw moistening, and this status was maintained throughout the experiment. The straw layer was also slightly moist for the structures S3 (soil/LFC/straw/HDPE) and S4 (soil/LFC/straw/10-cm air cushion/HDPE), but this phenomenon was more persistent for the former structure. Since the structure S4 has provided a proper balance between effective thermal protection and low moisture of the straw layer, this type of covering was considered the most appropriate concept for protecting overwintering snails, *C. aspersum*.

### 3.3. Testing of Protective Structure under Realistic Farm Conditions

**Figure 2:**
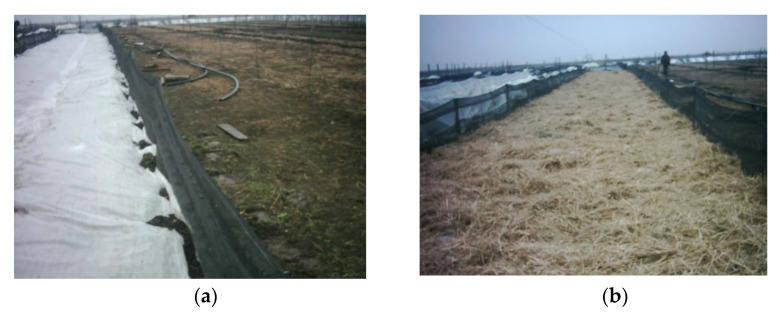

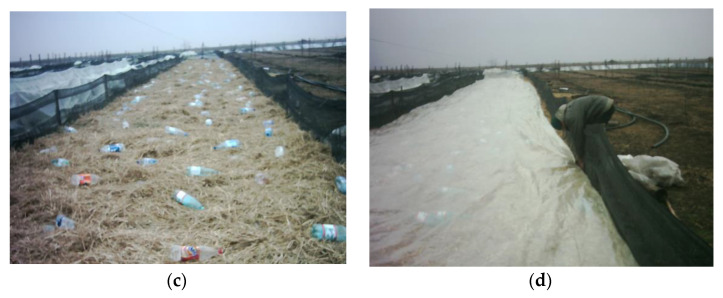
Installation of the “sandwich” system (**a**) Pens covered with a sheet of LFC. (**b**) Layer of straw over LFC. (**c**) PET bottlesabove the layer of straw. (**d**) Structure covered with HDPE sheet.

Based on the previousresults, we developed the “sandwich” system—named after its tri-stratified structure. Different steps of installing this system are shown in Figure 2a–d. The first step in this process was to create a network of micro shelters (e.g., ridge tiles, wooden pallets, timber slabs) just above the groundon at least half of the enclosure area. The pens were covered with a sheet of LFC that was anchored on all edges using a discontinuous layer of soil (Figure 2a). A loose layer of straw, 10/15 cm thick, was next put over LFC (Figure 2b). Finally, the structure was covered with a HDPE sheet (Figure 2d) suspended 5 to 10 cm above the layer of straw by using boxboards and PET bottles (Figure 2c).

**Figure 3:**
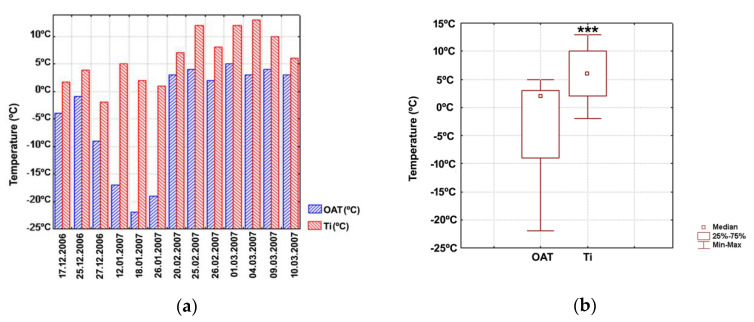
(**a**) Absolute values of ambient temperature and temperature for the pen protected with the “sandwich” system at selected time points. Data are shown as columns. (**b**) Median values for ambient temperature and temperature for the pen protected with the “sandwich” system. Data are shown as medians (point), with lower and upper quartiles (box) and minimum and maximum values (error bars). Marked boxes (*) indicate significant differences than the ambient temperature (Wilcoxon tests, ***—*p* ≤ 0.001, **—*p* ≤ 0.01, *—*p* ≤ 0.05). Ti, temperatures inside the “sandwich” system; OAT, ambient temperature.

Unlike pilot testing conducted in the village of Sâmbata de Sus, the area protected during this stage in the village of Muntenii de Sus was equal to the surface of a breeding pen (≈180 m^2^). Outdoor and indoor temperatures determined at selected time points are illustrated in Figure 3a. Night temperatures between 28 December 2004 and 15 February 2005 were generally below −5 °C, whereas diurnal values were rarely above +5 °C. The median values calculated based on the data collected at 13 time points, including the lower and upper quartiles and the minimum and maximum values, are shown in Figure 3b. Temperatures inside the “sandwich” system were significantly higher than ambient temperature (Figure 3b). We also note that differences in median temperatures measured inside and outside the protection system were higher in Muntenii de Sus than in pilot experiments conducted in the village of Sâmbata de Sus.

Data related to snail survival after hibernation and shell condition of dead specimens are given in Table 3. The 2006–2007 winter in Cugir and Ezeriş showed normal temperatures, with seldom moderate frosts and minimum temperatures up to −15 °C. For the farm located in Muntenii de Sus, there was also a normal winter pattern, with frequent moderate frosts and minimum outdoor temperatures up to −25 °C. Irrespective of location, the soil beneath the protective structure has never frozen. We identified significant differences in the survival of adult snails, *C. aspersum*, during the winter months (chi^2^ test, *p* < 0.001). Post hoc testing revealed significantly higher survival rates for pens protected using the “sandwich” system versus the pen protected using only LFC (Table 3). However, survival was similar among the pens using the “sandwich” system (Table 3).

There were also significant differences in the distribution of shell condition classes (chi^2^ test, *p* < 0.001). Post hoc analysis revealed significant differences when comparing the enclosures protected with the “sandwich” system to those protected using LFC (Table 3). Most shells of dead snails were intact, whereas the lowest frequency was observed for the shells with small holes (Table 3). The proportion of shells with big holes or smashed were within a close range at most locations (Table 3).

**Figure 4:**
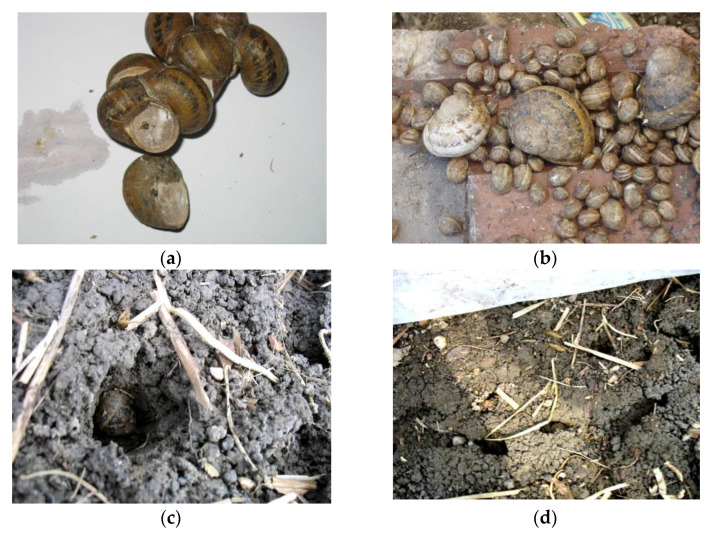
Selected behavioral aspects of overwintering *C. aspersum* (**a**) Overwinteringgregariousness in *C. aspersum*. (**b**) Adult and juvenile gastropods overwintering attached on the lower surface of ridge tiles. (**c**) Adult snail, *C. aspersum*, burrowed into the upper topsoil layer. (**d**) Network of tunnels dug by mature gastropods.

Field surveys conducted in winter 2006/2007 revealed interesting data about behavioral aspects of *C. aspersum* hibernation under ambient conditions provided by the “sandwich” system. A part of these habits isillustrated in Figure 4a–d. Although the resting position was not recorded when dismantling the protection system (March/April), we noticed that the vast majority of mature snails were still hibernating. In contrast, many juvenile individuals, and especially those having shell diameter less than 1 cm, were active at this time point. Adult gastropods showed a tendency towards overwintering under micro shelters, attached on their lower surface in large aggregations of up to 40 juveniles and 25 mature snails (Figure 4a,b). Other individuals were seen spending the winter with their aperture facing upwards, burrowed deep in the leaf litter above the soil or into the upper topsoil layer (Figure 4c). These habits had a lower frequency but were not uncommon. However, gastropods overwintering singly were also seldomly observed.

In loose sandy soils, mature gastropods occasionally dug a network of tunnels at the joint between leaf litter and the top 5 cm soil layer (Figure 4d), which covered up to 3–4 m^2^ and included smaller galleries (Figure 4d), wherein specimens of different sizes hibernated together. This behavior began to occur in September or October when the photoperiod was below 10 h, and nighttime temperature frequently fell below +4 °C. Although not within the purpose of our study, we also noted that juvenile gastropods tended to enter hibernation two to three weeks aftertheir mature counterparts. In addition, many of these sub-adult individuals were already active when dismounting the “sandwich” system.

## 4. Discussion

The present study results expand our knowledge of behavioral aspects of land snails during (pre)hibernation, which until now was mainly related to the ethology of wildpopulations [25,26,27,28,29,30,31,32,33,34]. The present paper also proposes a new method for outdoor hibernation of mature snails, *C. aspersum*, under humid temperate continental climate conditions. Understanding these behavioral aspects coupled with applying the “sandwich” system could serve as a step forward for the successful rearing of *C. aspersum* in open pastures in areas with colder winters than those from its native habitats.

### 4.1. C. aspersum (Pre)hibernal Behavior

In response to mixed action of low temperature and decreasing photoperiod, the brown garden snail, *C. aspersum*, starts to prepare for hibernation [4], with some specimens burying themselves in the ground or the leaf litters, while others are taking refuge under logs, boards, and stones [35]. Here, mature gastropods showed a significantly lower preference towards micro shelters built from iron sheets than for those built from either wood or ridge tiles, but a similar preference towards the latter two types of micro shelters. These findings could be related to different thermal properties of these materials, with wood and ceramics acting—in contrast to metals—as thermal insulators [36].

Consistent with other studies [19,37,38], we found that *C. aspersum* adults in preparation for overwintering can display burrowing behavior. Soil texture significantly influenced this behavior, with the percentage of burrowing gastropods being two-fold higher in the case of looser soil. The most plausible explanation is that these soils provide easier material to dig, thus facilitating burrowing by gastropods [39].

However, the burrowing snails did not migrate deep into the substratum, only burrowing down to 5 cm. The burrowing depth hence appears to be independent of the influence of soil texture. These data suggest that mature snails, *C. aspersum*, may act as opportunistic, shallow-burrowers rather than deep-burrowers before entering hibernation. This is in contrast with the behavior of other edible land snails of commercial importance. For example, the green snail, *Helix aperta* (Born, 1778), can burrow itself into the ground as deep as 15 cm before overwintering [40,41]. This behavior has also been observed and studied in other commonly farmed snail species, such as *Helix lucorum* (Linnaeus, 1758) or *Helix pomatia* (Linnaeus, 1758) [42,43].

### 4.2. Identification of Protection System (Pilot Level)

Lutrasil frost cloth (LFC) is dense enough to keep warmth inside and has a microporous structure allowing rain to pass through and reach the soil [24]. Designed to protect plants against frost and pests [24], this high-performance membrane is also used to provide winter protection for *C. aspersum* in farms using the ISISF system [8]. All protective structures tested in this study contained LFC, with temperatures measured under these coverings being significantly increased compared to ambient temperature. Although the structure S2 displayed the highest median temperature, it also contained the most moist straw. This latter observation, which may be linked to the infiltration of snowmelt water through LFC, does not favor its use for protecting overwintering snails. Thus, the humid temperate climate of Central Eastern Europe is characterized by moderate to abundant precipitation levels (rain, snow) and high day/night thermal amplitude during late winter to early spring, leading to frequent snowfall/snowmelt periods and freeze/thaw cycles within this period [44]. Such events can result in an increase in straw moisture content [45]. On one hand, this can decrease the thermal insulation capacity of the straw layer [45], thus affecting the thermal performance of the protective structure.On the other hand, excess moisture coupled with periods of warmer weather could promote premature awakening from hibernation [4,37], making the awoken/active snails particularly vulnerable to early spring freeze [4,8]. Moreover, shorter and/or interrupted hibernation has been linked to lower survival rates in juvenile snails [46] and lower overwintering survival and decreased fertility in adult gastropods [8,13,47].

The use of HDPE instead of LFC as the upper layers for structures S3 and S4 was associated with lower amounts of moist straw thanfor structure S2. HDPE is known to block oxygen transfer and promote water condensation on the inside surfaces when applied tothe soil [24]. Longer persistence of straw moistening observed for the structure S3 is likely the result of decreased ventilation resulting from applying the HDPE sheet directly on the straw layer.

### 4.3. Testing of Protective Structure under Realistic Farm Conditions

The thermal performance that a greenhouse provides overwinter is directly associated with the size of the covered area [48]. This may, at least partly, help explain the higher thermal performance seen when using the “sandwich” system under farm conditions. The fact that the soil surface did not freeze when using this protective structure supports the potential use of this concept for other purposes, such as the protection of early spring vegetables.

Compelling evidence points to the survival of mature specimens during winter acting as a crucial factor in the population dynamics of both wild gastropod populations [15,32,49,50] and farm-reared snails [4,12,13,43]. Application of the “sandwich” system yielded a significant improvement in overwinter survival compared to the sole use of LFC. In addition, it allowed higher survival rates compared to winter 2005/2006, with over two-thirds of the adult snails, *C. aspersum*, successfully passing the winter. Moreover, this death rate is below the normal range of mortalities in areas with milder winters, such as France, Turkey, or Southern California, whichis ~50–80% [13,37,51].

Unlike the native Roman snail *Helix pomatia*, the brown garden snail, *C. aspersum*, has a limited ability to cool and survive ice formation in its tissues, being considered a partially freezing tolerant species. This gastropod can survive to below-zero temperatures for a relatively short period, amaximumof 9.8 h for exposure to temperatures as low as −5 °C [34]. To the best of our knowledge, there are no confirmed data in malacological literature about the presence of wild populations of *C. aspersum* in Romania despite its success in colonizing new habitats [9,52]. In fact, stable populations have been reported only for limited areas in Central Eastern Europe, such as in Austria near Vienna [53,54]; in Hungary in areas like Tihany, Vecsés, Ercsi, and Monor [9]; or in Bohemia near Praga [18]. Moreover, this terrestrial gastropod is found in Scotland only near the seaside, where freezing occurs more rarely than in the inner lands [35]. Taken together with our findings, these data render the cold winter weather being a limiting factor for *C. aspersum* spread in a continuous areal in CentralEastern Europe.

Most shells of dead individuals were intact, and therefore, the potential causes of death may only be guessed: diseases, parasites, cold weather, premature arousal, starvation, and physical stress may all have been involved. Given the comparable distributions of this shell condition class across the pen(s) protected with and without the “sandwich” system, one can infer that cold weather may be, to some extent, responsible for the observed mortalities. The frequencies of smashed shells and shells with large holes were within a close range for all pens, lending support for rodents (and other small mammals) as the main biotic factor affecting overwinter survival in investigated farms. This is consistent with data from other studies [19,37]. However, using straw appears to have a small effect on the rodent-related death rates, as suggested by comparable percentages of the foregoing shell condition class in enclosures with or without straw. Furthermore, the relatively small number of shells with small holes implies that invertebrate predators [37] were minor factors affecting overwinter survival.

Juvenile snails with shell diameters less than 1 cm tended to enter later and awake earlier from hibernation than their mature counterparts. Indeed, individuals of similar age and size are known to display this habit and be active throughout the warmer winter periods [37]. This behavior is likely to affect the overwinter survival of *C. aspersum* juveniles, especially in Similar differences in entering and exit time, as well as the shorter duration of hibernation of juvenile snails, have been reported for wild populations of several snail species, which undergo winter hibernation, such as *Helix lucorum* (Linnaeus, 1758) [55] and *Cepaea vindobonensis* (Ferrusac, 1821) [56].

We also note that the length of hibernation was more than five months. This duration is close to those reported for the Atlantic coast of France, longer thanthose observed in Spain, but shorter than those identified in Scotland or Wales [57]. Under this framing, metabolic adaptions needed for lengthening the hibernation period may serve as another factor limiting the ability of this species to inhabit colder latitudes [57].

Although the burrowing behavior was not uncommon, most snails tended to hibernate clustered together, primarily attached to the lower surface of micro shelters and secondarily at the soil surface. When the protection system was dismantled, the highest survival tended to occur for individuals hibernating in large aggregations. This association between higher survival and overwintering gregariousness could indicate that these specimens have found particularly sheltered locations to take refuge during winter, where they may be protected against the direct impact of temperature, wind, or humidity [32]. Snail aggregation during hibernation may also help to reduce total exposed surface area, maintain body water, preserve heat, and/or conserve energy during winter [32,46], although alternative benefits, such as reduced predation risk, cannot be excluded [58,59].

## 5. Conclusions

Mature gastropods in preparation for hibernation exhibited a significantly higher preference for wood and ridge-tile micro shelters than for corrugated iron micro shelters. Soil texture significantly influenced the burrowing behavior, but not the burrowing depth. All pilot structures tested displayed significantly higher thermal protection efficiency compared to the sole use of LFC. The balance between straw moistening and thermal protection favored using structure soil/LFC/straw/10-cm air cushion/HDPE—the “sandwich” system. Under farm conditions, snails tended to hibernate clustered together, attached to the lower surface of micro shelters. The use ofthe “sandwich” system yielded significantly higher thermal protection efficiency and snail survival thanthe sole use of LFC. Predator occurrence appeared to affect overwintering survival only marginally. These data suggest that the combined use of the “sandwich” system and ridge-tile/wood micro shelters could be an effective solution for overwintering mature *C. aspersum* snails in colder climates.

## Figures and Tables

**Table 1:** Distribution of snail classes concerning their preference for micro shelters investigated.

Snail Class	Type of Micro Shelter		
	Roofing Sheets	Ridge Tiles	Wooden Pallets
Attached snails	22% (11)	56% (28) ***	50% (25) **
Unattached snails	78% (39)	44% (22)	50% (25)

Data for the distribution of gastropods among different preference classes are shown as percentages with absolute values (in parentheses). Marked values (*) indicate significant differences as compated to the use of corrugated roofing sheets as micro shelters (Chi^2^ tests based on 2 × 2 contingency tables, ***—*p* ≤ 0.001, **—*p* ≤ 0.01, *—*p* ≤ 0.05).

**Table 2:** Distribution of snails across different classes related to the burrowing behavior.

Snail Class	Site ST1	Site ST2
Burrowed snails	34% (34) **	17% (17)
Non-burrowed snails	66% (66)	83% (83)
Snails burrowed within the top 5 cm soil layer	90.20% (30.66)	94.12% (16)
Snails burrowed deeper than 5 cm in the soil	9.80% (3.33)	5.88% (1)
Snails attached to micro shelters	62.12% (41)	57.83% (48)
Snails lying on the ground	37.88% (25)	42.17% (35)

Data for the distribution of gastropods among different burrowing classes are shown as percentages with mean absolute values (in parenthesis). These values represent the means for three replicate experiments. Marked values (*) indicate significant differences between site S1 and site S2 (Chi^2^ tests based on 2 × 2 contingency tables, ***—*p* ≤ 0.001, **—*p* ≤ 0.01, *—*p* ≤ 0.05). Site ST1, the village of Urecheşti (Gorj county); site ST2, the village of Banloc (Timiş county).

**Table 3:** Distribution of snail survival classes for different types of “sandwich” systems.

Snail Class	Site ST3	Site ST4	Site ST5	Reference Pen
Alive snails	70.99% (6283) ***	68.07% (5675) ***	67.81% (8011) ***	47.60% (476)
Dead snails	29.01% (2567)	31.93% (2662)	32.91% (3803)	52.40% (524)
**Shell condition**				
Intact shell	64.43% (1654) ***	47.60% (1267) *	67.95% (2584) ***	53.05% (278)
Large hole	13.21% (339) ***	20.40% (543)	21.33% (811)	23.66% (124)
Smashed	13.79% (354)	15.93% (424)	8.73% (332) ***	15.26% (80)
Small holes	8.57% (220)	16.08% (428) ***	2.01% (7) ***	8.01% (42)

Data for the distribution of different survival classes and shell condition classes are shown as percentages with absolute values (in parenthesis). Marked values (*) indicate significant differences compared to the reference pen (Chi^2^ tests based on 2 × 2 contingency tables, ***—*p* ≤ 0.001, **—*p* ≤ 0.01, *—*p* ≤ 0.05). Site ST3, the city of Cugir (Alba county); site ST4, the village of Ezeriș (Caraş-Severin county); site ST5, the village of Muntenii de Sus (Vaslui county).

## Data Availability

All relevant data are within the paper.
